# One-pot synthesis of CuPt nanodendrites with enhanced activity towards methanol oxidation reaction†

**DOI:** 10.1039/c8ra00391b

**Published:** 2018-03-05

**Authors:** Hongcheng Peng, Weihong Qi, Haofei Wu, Jieting He, Yejun Li, Haipeng Xie

**Affiliations:** School of Materials Science and Engineering, Central South University Changsha 410083 P. R. China; Key Laboratory of Non-ferrous Materials Science and Engineering, Ministry of Education Changsha 410083 P. R. China; Hunan Key Laboratory of Super Microstructure and Ultrafast Process, School of Physics and Electronics, Central South University Changsha Hunan 410083 China qiwh216@csu.edu.cn yejunli@csu.edu.cn

## Abstract

CuPt nanodendrites have special electrocatalytic performance due to their unique morphologies and structures. In this work, we report a facile and one-pot synthesis of highly branched and dispersed CuPt nanodendrites with a size of about 22 nm and Pt/Cu ratio of 2 : 1. X-ray photoelectron spectra indicate a Pt-rich surface of the prepared CuPt nanodendrites. The combination of a highly branched structure with a Pt-rich shell would be very beneficial for catalytic properties, which was confirmed by electrochemical tests towards MOR. A systematic study on morphology evolution of the products and understanding of their growth mechanism was carried out using the effects of reaction time and reducing agents.

## Abstract 2

CuPt nanodendrites have special electrocatalytic performance due to their unique morphology and structures.

## Graphical abstract

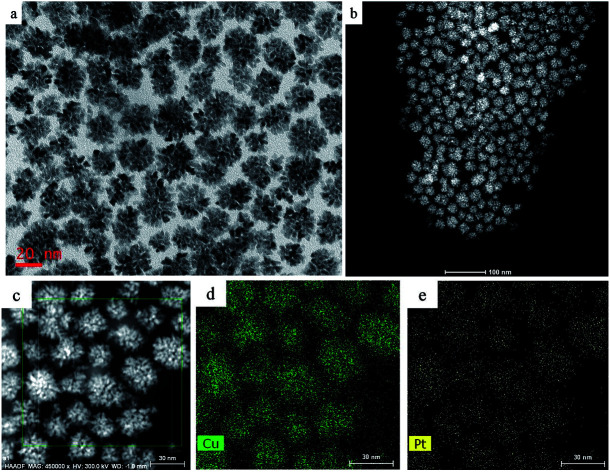

## Introduction

Pt and its alloys play an important role as catalysts in electrochemical and chemical reactions.^1–9^ For the properties of low-dimensional solids depending on crystal size and shape,^10^ a large surface-to-volume ratio further improves the catalytic properties of Pt-based nanoparticles. Studying Pt-based bimetallic nanoparticles is not only economical due to the price of Pt metal, but also because they show improved catalytic activity due to the bi-functionality of both components. Moreover, the morphology of nanoparticles is critical to their physical and chemical performances due to the different surface lattice faces of various morphologies. Hence, controlled-morphology synthesis of Pt-based bimetallic nanoparticles have attracted wide research interests.

Recently, nanoparticles with a highly branched structure, called nanodendrites, have attracted great attention. Unlike polyhedral nanoparticles, nanodendrites have greater potential as catalysts due to their unique and anomalous structures, high surface-to-volume ratios, and rich edges and corners.^11^ Some branched monometallic nanoparticles, such as Pt,^12–16^ Pd,^17–19^ Au,^20^ and Ag,^21^ have been achieved successfully. Controlled synthesis of branched Pt-based bimetallic nanocrystals, in particular CuPt nanodendrites, with tunable size and structure has demonstrated a great potential to advance their electrocatalytic performances toward methanol oxidation (MOR), oxygen reduction reaction (ORR), and so on.^4,5,8,22–25^ Recently, Taylor *et al.*^22^ reported a two-step co-reduction synthesis of CuPt nanodendrites (30 nm), which involved a kinetically controlled slow reduction step and, subsequently, a fast reduction process, where the size and composition of the CuPt nanodendrites increased with the reaction time. A facile synthesis of CuPt bimetallic alloy nanodendrites (∼35 nm) using Brij 58 as a template was investigated by Fu *et al.*^23^ The highly branched structures and porous features offer relatively large surface areas, which greatly enhanced the catalytic activity for ORR. A modified polyol reduction of CuPt hierarchical branched nanoparticles (∼30 nm) by a coefficient of poly-(vinylpyrrolidone) (PVP) and HCl was studied by Cao *et al.*,^24^ where a synthesized sample with open porous structure exhibited greatly enhanced activity toward MOR. Guo *et al.*^25^ reported a rapid aqueous synthesis of CuPt nanodendrites with tuning the branches and composition *via* the underpotential deposition of Cu, where the influence of different reaction conditions on the formation of CuPt nanodendrites, *i.e.*, size, morphology, and the composition, were systematically investigated. Interestingly, they found that Pt_55_Cu_45_ nanodendrites with smaller size (∼14 nm) and less branches but higher content of Cu exhibited the highest electrocatalytic activity for both ORR and MOR. Aside from the improved catalytic behaviors, in general, different from the synthesis of pure metal nanocrystals, the coexistence of two precursors for CuPt nanodendrites will complicate the synthesis process, especially in a one-pot synthetic strategy. Therefore, more efforts need to be devoted to the understanding of the morphology-controlled synthesis of branched Pt-based bimetallic nanoparticles and their related electrocatalytic properties.

This paper reports a facile and one-pot synthesis method developed to prepare CuPt nanoparticles with a highly branched structure. The products have uniform size and morphology, which are dispersed with clear branched structures. The growth mechanism of CuPt nanodendrites was investigated, which showed that reducing agents play significant roles on morphology of the prepared samples. Interestingly, the X-ray photoelectron spectra indicate a Pt-rich surface of the prepared CuPt nanodendrites. A combination of the highly branched structure with Pt-rich surface would be very beneficial for catalytic properties, which is confirmed by electrochemical tests towards MOR.

## Experimental details

### Materials

All chemicals in this synthesis were of analytical reagent grade. Chloroplatinic acid hexahydrate (H_2_PtCl_6_·6H_2_O), copper(ii) chloride dihydrate (CuCl_2_·2H_2_O), poly(vinylpyrrolidone) (PVP, MW = 55 000), ethylene glycol (EG), oleic acid, oleic acid, and hydrazine hydrate were purchased from Aladdin Industrial Inc. (Shanghai, China). Vulcan XC-72 was purchased from Cabot (Boston, MA, USA). Nafion (5%) was purchased from Sigma (Shanghai, China). All materials were used as received without further purification.

### Preparation of Cu-Pt nanodendrites

In a standard synthesis, 0.5 mmol CuCl_2_·2H_2_O, 1 mmol PVP, and 2.5 mmol ascorbic acid were dissolved in 10 mL of EG in a three-necked flask under a N_2_ stream. The mixture was pre-heated to 80 °C and refluxed under magnetic stirring for 30 min. Then, 0.2 mmol H_2_PtCl_6_·6H_2_O solution was dropped into the flask with a pipette. The reaction temperature was increased to 140 °C for 1 h and then cooled to room temperature. The products were collected *via* centrifugation and washed several times using ethanol and deionized water. Then, the obtained CuPt nanodendrites were dispersed in ethanol.

### Preparation of Cu-Pt nanodendrites/Vulcan XC-72

A given weight of Vulcan XC-72 was mixed with the as-prepared solution of CuPt nanodendrites for an hour. The mixture was collected *via* centrifugation (12 000 rpm, 10 min) and further purified twice with deionized water and ethanol, then dried in a vacuum oven at 80 °C.

### Characterization

Transmission electron microscopy (TEM), high-resolution transmission electron microscopy (HRTEM), and high-angle annular dark-field scanning transmission electron microscopy (HAADF-STEM) images were obtained using an FEI Tecnai G2 F20 transmission electron microscope. The products were dropped on copper grids coated with an amorphous carbon film for TEM. Scanning electron microscopy (SEM) images were obtained using an FEI Helios NanoLab 600i scanning electron microscope. Powder X-ray diffraction (XRD) analysis was taken by a Siemens D500 diffractometer. An X-ray photoelectron spectroscopy (XPS) spectrum was obtained on a SPECS ultrahigh vacuum system.^26^

### Electrocatalytic activity valuation

Electrocatalytic activities of the CuPt nanodendrites/C were characterized using cyclic voltammetry (CV) and current–time (*i*–*t*) measurements on a CHI660D electrochemical workstation in a three-electrode configuration with a saturated calomel electrode (SCE) and a platinum foil as the reference and counter electrodes, respectively. The catalyst suspension was prepared by mixing CuPt nanodendrites/Vulcan XC-72 (2 mg), ethanol (0.75 mL), deionized water (0.25 mL), and Nafion solution (5%, 50 μL), and was then spread onto a glassy carbon (GC) electrode. A commercial Pt/C (30 wt% Pt) catalyst was also tested for comparison. CV measurements were performed in 0.5 M NaOH solutions under a flow of N_2_ at a sweep rate of 50 mV s^−1^. The MOR was performed in a solution containing 0.5 M CH_3_OH and 0.5 M NaOH at a scan rate of 50 mV s^−1^.

## Results and discussion

We performed a standard kinetically controlled synthesis of Cu-Pt nanodendrites. At the initial stage, a portion of Cu precursors were reduced *via* ascorbic acid as the reducing agent in the EG solution at low temperature. Meanwhile, N_2_ was flowed into the system to provide a protective atmosphere. Over 30 min, the reaction solution turned to light brown. In the next step, the Pt precursors were added into the system and the reaction temperature immediately was increased to 140 °C. The solution color turned from light brown to black within 1 h. In a standard synthesis, ascorbic acid as a strong reducing agent was added to control the morphology of CuPt nanoparticles by modifying the deposition and diffusion rate of metal atoms. We chose PVP as the surfactant because the N and O groups could adsorb to specific sites on the surface of metal atoms, blocking the crystal growth on those sites.^27–30^

**Fig. 1:**
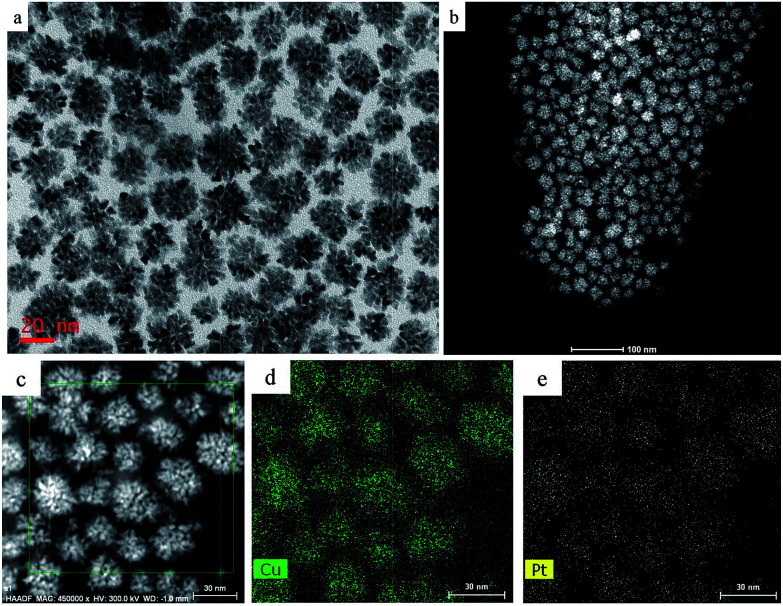
(a) TEM, (b) HAADF-STEM, and (c–e) EDS mapping images of the CuPt nanodendrites.

Typical TEM images show that the overall size of a sample's CuPt nanodendrites is ∼22 nm (Fig. 1). The TEM and STEM images (Fig. 1b) demonstrated that almost all the synthesized Cu-Pt particles present a highly branched morphology. (See Fig. S1† for more TEM images.) Fig. 1c–e show the HAADF-STEM and EDS elemental mapping images, which demonstrate a uniform distribution of Pt and Cu atoms in all of the nanoparticles.

**Fig. 2:**
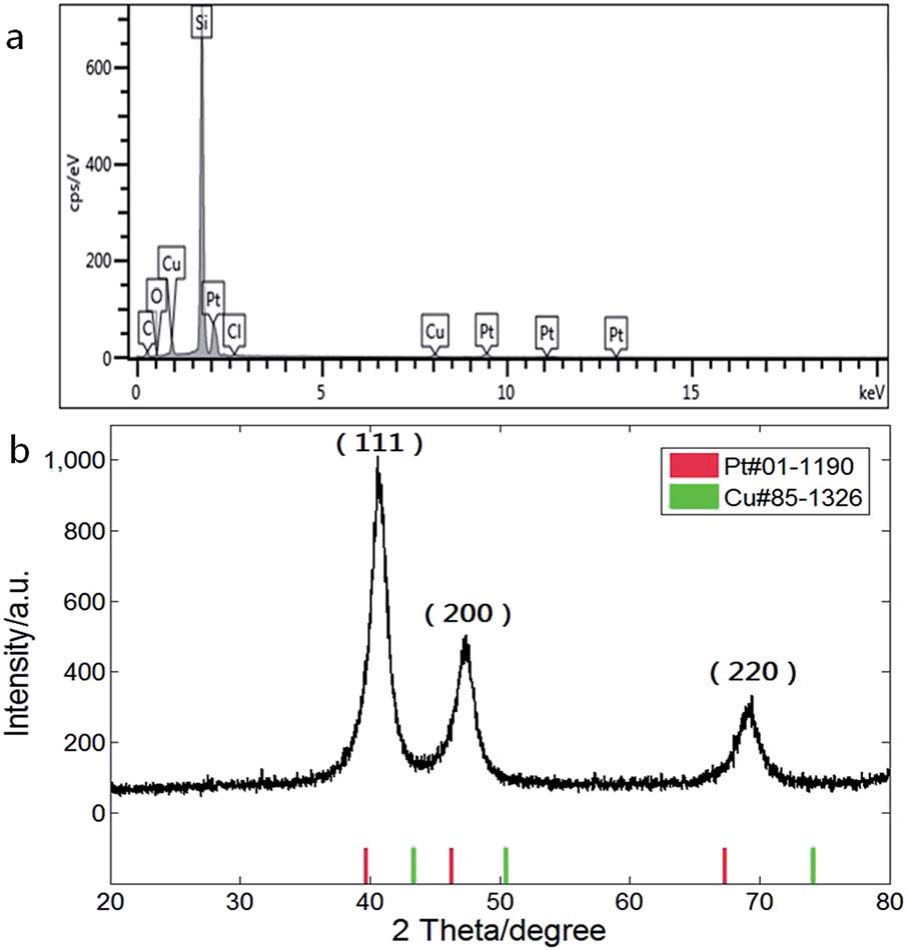
(a) Energy spectrum and composition analysis and (b) XRD pattern of CuPt nanodendrites.

Composition of the CuPt nanodendrites was measured by energy dispersive X-ray spectroscopy equipped on the SEM as shown in Fig. 2a, which revealed an atomic ratio between Pt and Cu of about 2 : 1. The XRD pattern (Fig. 2b) shows a typical fcc structure with characteristic peaks located at 2*θ* = 40.7°, 47.3°, and 69.3°, which correspond to (111), (200), and (220) planes, and lie between those of pure Pt (red) and Cu (green), indicating the formation of CuPt alloys. The lattice constant of the CuPt nanodendrites is estimated to be 0.381 nm *via* analysis of the XRD pattern. Based on Vegard's law, the Pt/Cu ratio was estimated as 1.8 (*a*_Pt_ = 0.392 nm, *a*_Cu_ = 0.361 nm), which is close to the result of EDS-SEM analysis.^31^

**Fig. 3:**
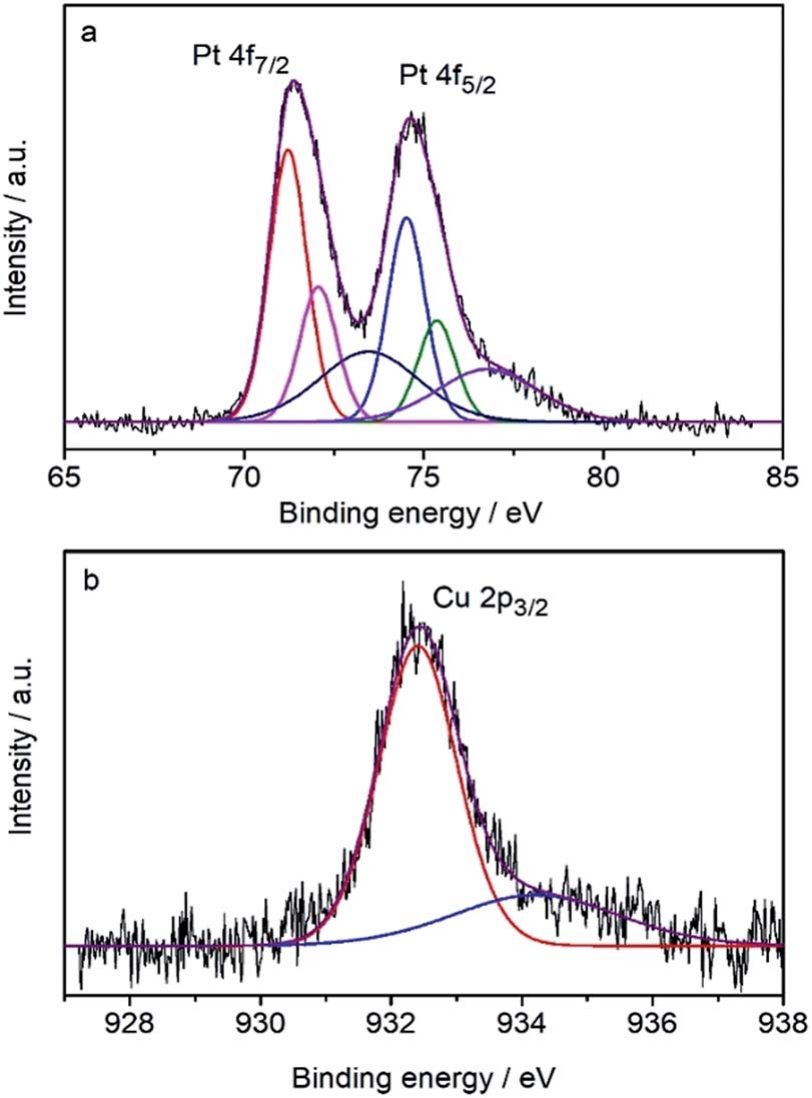
XPS spectra of (a) Pt and (b) Cu in the CuPt nanodendrites.

The surface composition was determined by the core-level X-ray photoelectron spectra (XPS) (Fig. 3) to be Pt_82_Cu_18_, which is Pt-rich compared with the composition of the CuPt nanodendrites. It shows that the Pt 4f_7/2_ peak (71.23 eV) is very close to that of pure Pt (71.2 eV), therefore the surface Pt was judged to be pure metallic atoms. Note the Cu 2p_3/2_ peak at 932.4 eV is shifted toward lower binding energy compared with the pure Cu data (932.7 eV), indicating the expansion of atomic distance because of the formation of an alloy with Pt atoms. These results suggest that CuPt nanodendrites are composed of a metallic Pt shell and an alloyed core.

**Fig. 4:**
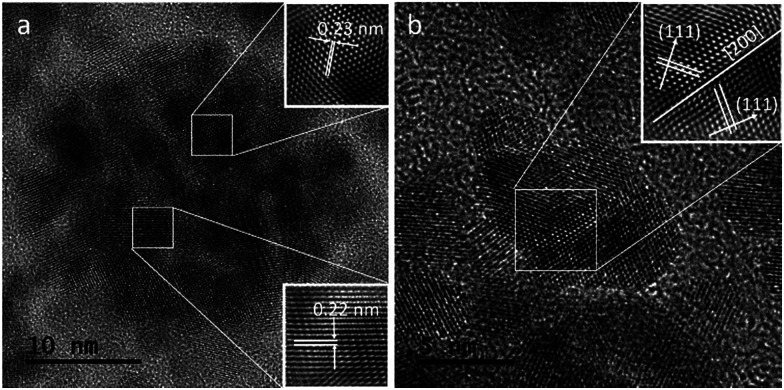
HRTEM images of the CuPt nanodendrites.

**Fig. 5:**
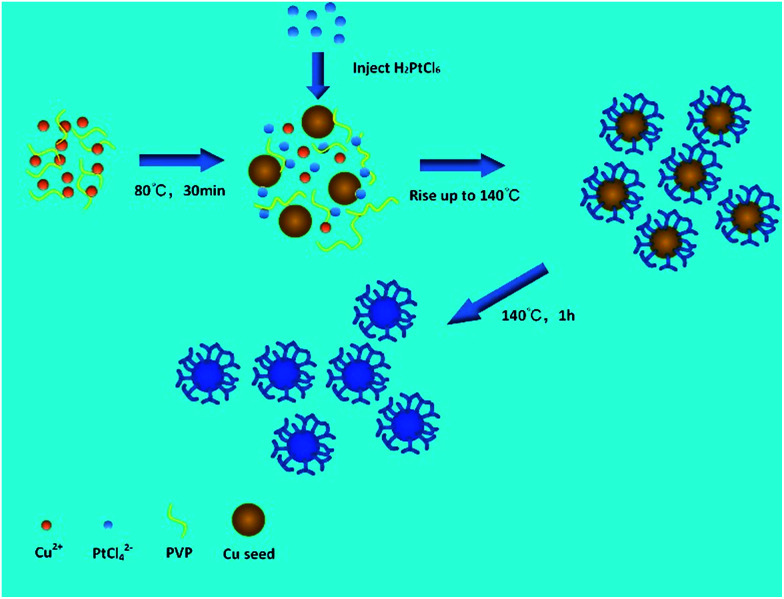
Schematic illustration of the growth mechanism of CuPt nanodendrites.

Fig. 4a shows a HRTEM image of a single CuPt nanodendrite. The lattice fringe of the nanodendrite is very clear, with an interfringe distance ∼ 0.23 nm corresponding to the (111) planes of pure Pt or PtCu alloy. Fig. 4b is the magnified image of a branch on the CuPt nanodendrites. It shows that the branch displays a double twins structure. The twins plane is (200), and the two twins grow along the 〈111〉 direction. So, this process can be described as a preferential overgrowth along the 〈111〉 direction. As shown in Fig. 5, the growth mechanism of the CuPt nanodendrites can be approximated as follows: at the first stage, the concentration of Cu^2+^ ions stayed high, a portion of Cu^2+^ ions were reduced to form Cu seeds. At low temperature (80 °C) as well as the decreased concentration of Cu^2+^, the reduction rate turned lower. Half an hour later, the Pt precursor was injected into the solution and Cu^2+^ and PtCl_4_^2−^ ions were intensely co-reduced along Cu seeds 〈111〉 direction by ascorbic acid. Since the reduction potential of Cu^2+^/Cu (0.340 V) is smaller than that of PtCl_4_^2−^/Pt (0.758 V), a portion of Cu^2+^ ion may be reduced and re-oxidized along with the reaction process,^24^ which may serve as deposition or nucleation sites for formation of more branches. Finally, the Cu and Pt atoms among CuPt nanodendrites diffused and formed nanoalloy. It should be noted that because of the easy oxidization of Cu, a portion of the Cu^2+^ ions remain,^32^ which results in a higher Pt/Cu ratio (2 : 1) in the obtained nanodendrites than the original ratio (2 : 5). This process conforms to the heterogeneous seeded growth mechanism^11^ instead of the rapid-growth mechanism.^33^ In Lim and Xia's theory, pre-synthesized nanocrystals serve as seeds for further growth of another metal with a different reduction process. The homogeneous nucleation and particles attachment also play considerable roles in the heterogeneous seeded growth. Due to the large surface-to-volume ratio and high collision frequency in the synthesis process, particles attachment is very likely to occur. The morphologies of particles are adjusted by the deposition and diffusion rate of metal atoms.

**Fig. 6:**
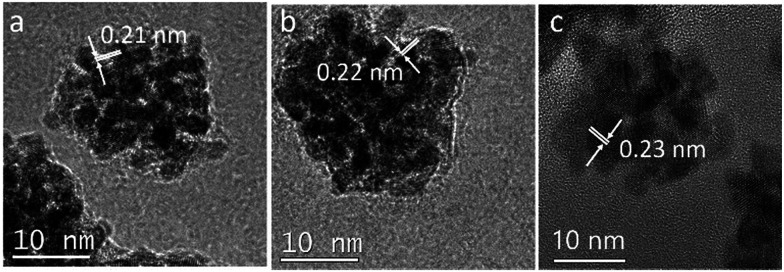
HRTEM images of CuPt nanoparticles synthesized under different reaction times: (a) 5 min, (b) 30 min, and (c) 1 h.

To further understand the morphology evolution of the formation of CuPt nanodendrites during the experimental process, a systematic study was conducted to investigate the effects of reaction time and reducing agents. Fig. 6 shows the HRTEM images of prepared CuPt nanoparticles at different reaction times. By comparing samples at reaction times of 5 min, 30 min, and 1 h, it is surprising to find that the CuPt nanoalloy has already taken the morphology of dendrites a few minutes after the reaction. With increasing reaction time, the size of CuPt nanodendrites grows from ∼18 nm (5 min), over ∼20 nm (30 min), to ∼22 nm (1 h), while the morphology stays the same. By studying the interfringe distance of the outer layer of the CuPt nanodendrites, it was found that the distance of (111) plane increased slightly from ∼0.21 to ∼0.23 nm. We assumed that this is due to the growth of the nanodendrites (more Pt than Cu as Cu is easily re-oxidized), as well as the diffusion and mixing of the Pt and Cu atoms to form an alloy.

**Fig. 7:**
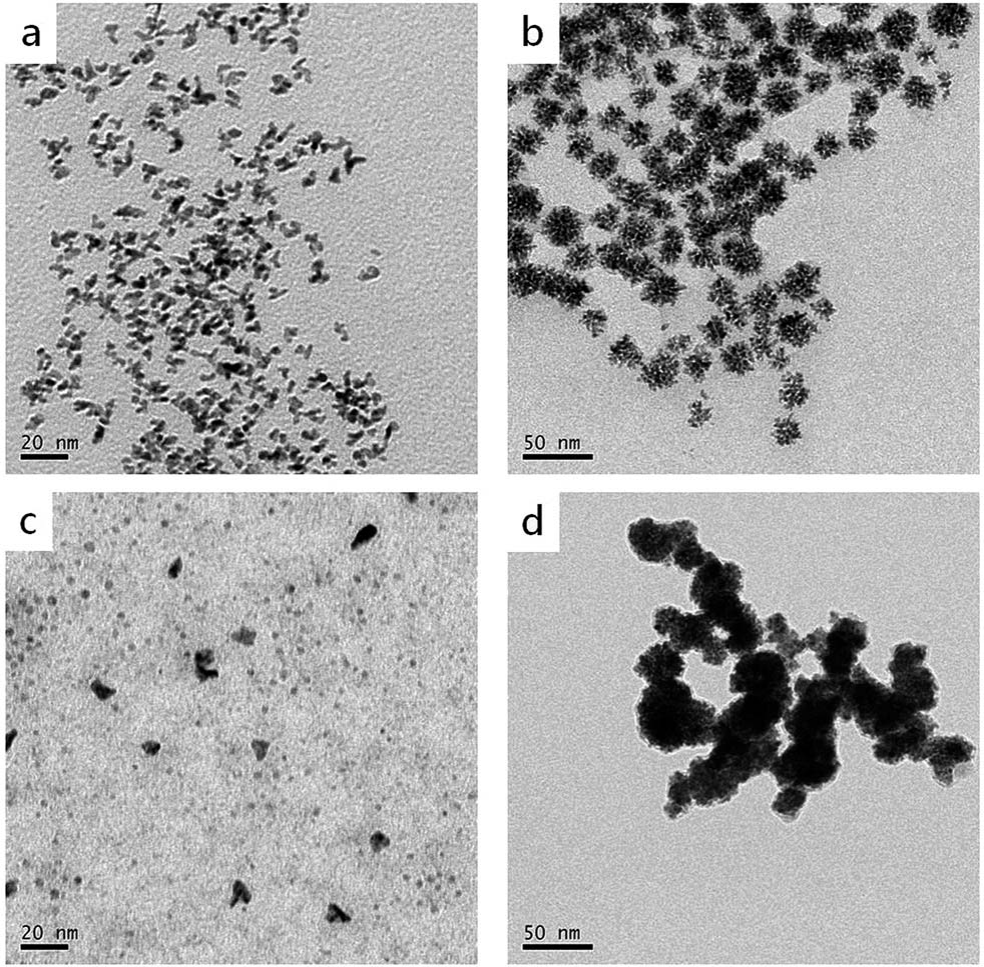
TEM images of CuPt nanoparticles synthesized under different reducing conditions: (a) 1.25 mmol ascorbic acid, (b) 5 mmol ascorbic acid, (c) 2.5 mmol citric acid, and (d) 2.5 mmol hydrazine hydrate.

As is known, reducing agents play significant roles in the formation of highly branched structures. A series of experiments were conducted to test the effect of the amount of ascorbic acid to the products. If the amount of ascorbic acid is decreased to 1.25 mmol, the products are composed of a large number of dispersed branches (Fig. 7a). When the reducing agent concentration is lower, the solution doesn't have enough reducing potential to grow sufficient Cu seeds in the first step (80 °C). When the temperature increased to 140 °C, a large number of Cu^2+^ and PtCl_4_^2−^ ions didn't have sufficient nucleation sites to grow; therefore, they formed monodispersed branched nanocrystals. When the ascorbic acid was increased to 5 mmol, the products displayed very similar morphology and size with the standard sample (Fig. 7b), indicating 2.5 mmol ascorbic acid is enough for the formation of branched nanodendrites during the reaction process. Furthermore, different reducing agents, *i.e.*, citric acid and hydrazine hydrate, were tested (Fig. 7c and d). In comparison with ascorbic acid, citric acid is a weak reducing agent; therefore, the Cu^2+^ and PtCl_4_^2−^ ions may not have sufficient nucleation rates to overgrow. At the end of the experiment, only small Pt, Cu, or alloyed particles were formed (Fig. 7c). On the contrary, hydrazine hydrate is a stronger reducing agent than ascorbic acid, even at a low temperature. Therefore, before by injecting Pt precursor, more Cu^2+^ ions were reduced, and Cu particles agglomerated. Then, the rest of Cu^2+^ and the injected PtCl_4_^2−^ ions were co-reduced to form larger and aggregated particles (Fig. 7d).

**Fig. 8:**
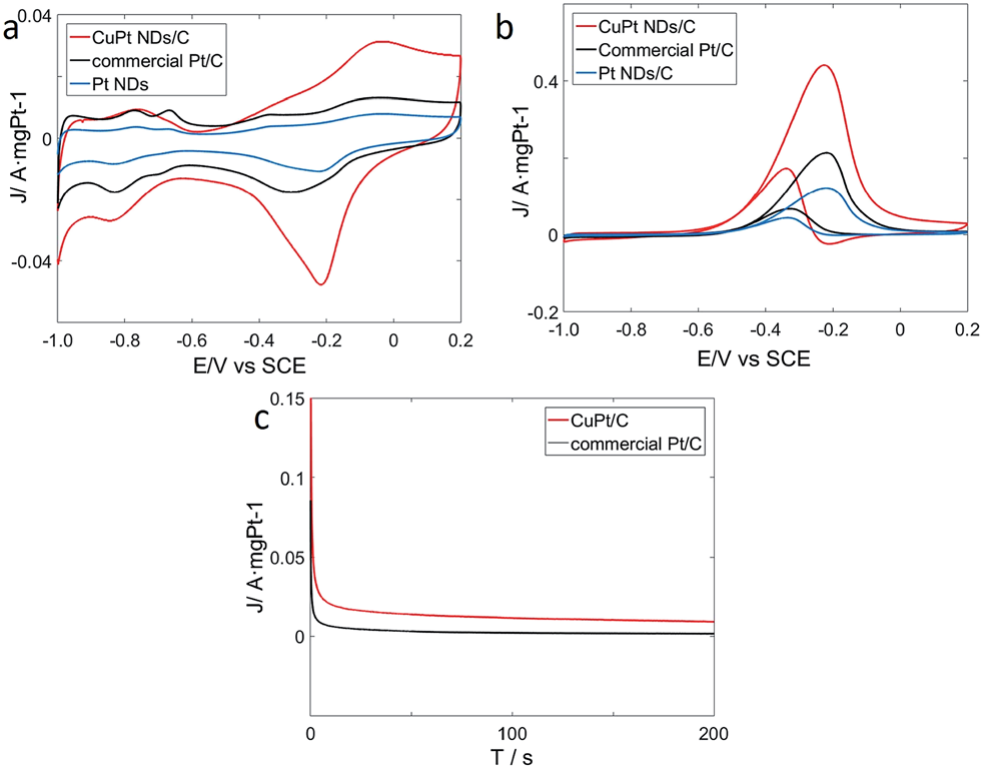
(a) Cyclic voltammetry (CV) curves of the catalysts in 0.5 M NaOH, (b) CV curves of the catalysts in 0.5 M NaOH and 0.5 M CH_3_OH, (c) current–time (*i*–*t*) curves of the catalysts in 0.5 M NaOH and 0.5 CH_3_OH at −0.32 V.

To explore catalytic properties, we tested the activity of the prepared CuPt nanodendrites towards MOR. The CuPt nanodendrites were loaded onto a Vulcan XC-72 carbon black support to minimize possible aggregation and utilize the support effect during the catalytic reaction. We measured cyclic voltammetry (CV) curves of CuPt nanodendrites/C, Pt nanodendrites/C, and the commercial Pt/C catalyst in a N_2_-purged 0.5 M NaOH aqueous solution at a sweep rate of 50 mV s^−1^ (Fig. 8a). CV curves of CuPt/Vulcan XC-72 (red line) reveal that the hydrogen adsorption–desorption peaks and Pt oxidation–reduction peaks are well observed. The hydrogen adsorption–desorption peaks of CuPt nanodendrites/C are clearly larger than those of commercial Pt/C and Pt nanodendrites/C, which demonstrates that the CuPt nanodendrites/C catalysts have a larger electrochemically active surface area (ESCA) and better catalytic performance.

The electrochemical performances of CuPt nanodendrites/C, Pt nanodendrites/C, and commercial Pt/C for MOR were tested *via* CV curves in 0.5 M NaOH and 0.5 M CH_3_OH (Fig. 8b). In the forward scan, the oxidation peaks were at approximately −0.23 V for both three catalysts, which is due to the direct oxidation of methanol adsorbed on the electrode surface. While in the backward scan, it was at around −0.34 V for CuPt nanodendrites/C and −0.32 V for commercial Pt/C, which is because of the removal of carbonaceous species that were not completely oxidized in the forward scan.^34^

As shown in Fig. 8b, the CuPt nanodendrites show a peak current (1.10 A mg^−1^) for MOR that is 2 times higher than that of commercial Pt/C catalysts (0.54 A mg^−1^), and 3.7 times higher than that of pure Pt nanodendrites/C catalysts (0.30 A mg^−1^). Those results also suggest that the CuPt nanodendrites have a better tolerance towards CO poisoning. MOR measurements were also carried out for the products with different reaction times and reducing agents (see Fig. S2a and b†), which in general show worse catalytic performance. The poor MOR performance of products with citric acid is due to the formation of small Pt and Cu particles, while that with hydrazine hydrate is because of the strong agglomeration of the nanoparticles. Although the products at 5 min and 30 min with ascorbic acid show similar morphology to the standard CuPt nanodendrites with 1 h, their weak MOR reactivity can be because the Cu and Pt atoms do not have enough time to diffuse and form nanoalloy.

To further understand the electrochemical durability performances of the prepared catalysts, current–time (*i*–*t*) curves were measured in a solution of 0.5 M NaOH and 0.5 M CH_3_OH at −0.32 V (Fig. 8c). The curves of the two catalysts declined rapidly at the initial stage, which is due to the formation of CO-like intermediates, such as CO and CHO.^35,36^ Later, these CO-like intermediates will adsorb on the surface of active Pt atoms to prevent further methanol oxidation. After that, the two curves continue to decrease and gradually achieve a stable state. In general, it is indicated that the CuPt nanodendrites/C catalysts show better stability than commercial Pt/C.

## Conclusions

In summary, a facile synthesis of highly branched CuPt nanodendrites was developed. The prepared CuPt nanodendrites were well dispersed with a size distribution of about 22 nm. A set of experiments was carried out to study the influence of reaction time and reducing agents on the morphology evolution of the products. It was surprising to find that the CuPt nanoalloy had already taken the morphology of dendrites a few minutes after the reaction. The weak citric acid or strong hydrazine hydrate can only result in smaller or larger particles, with no formation of branched nanodendrites. Because of the highly branched morphology as well as the Pt-rich shell structure, the synthesized CuPt nanodendrites showed better activity and CO-tolerance toward MOR than that of commercial Pt/C catalysts.

## Conflicts of interest

There are no conflicts to declare.

## Supplementary Material

RA-008-C8RA00391B-s001
