# Supplementary material for: One-pot synthesis of CuPt nanodendrites with enhanced activity towards methanol oxidation reaction
Source: RSC Adv. 2018 Mar 5;8(17):9293–8. doi: 10.1039/c8ra00391b (PMC9078692; doi:10.1039/c8ra00391b)
Supplement: RA-008-C8RA00391B-s001 [file RA-008-C8RA00391B-s001.pdf]

Electronic Supplementary Material (ESI) for RSC Advances.
This journal is © The Royal Society of Chemistry 2018

Supplementary Information

One-Pot Synthesis of Cu-Pt Nanodendrites with Enhanced Activity towards Methanol Oxidation Reaction

Hongcheng Peng[1], *Weihong Qi*[1, 2, *], *Haofei Wu*[1], *Jieting He*[1], *Yejun Li*[3*], and *Haipeng Xie*[3]

[1] School of Materials Science and Engineering, Central South University, Changsha, 410083, P. R. China

[2] Key Laboratory of Non-ferrous Materials Science and Engineering, Ministry of Education, Changsha, 410083, P. R. China

[3] Hunan Key Laboratory of Super Microstructure and Ultrafast Process, School of Physics and Electronics, Central South University, Changsha, Hunan 410083, China

[*] **Corresponding author**: qiwh216@csu.edu.cn; yejun.li@csu.edu.cn

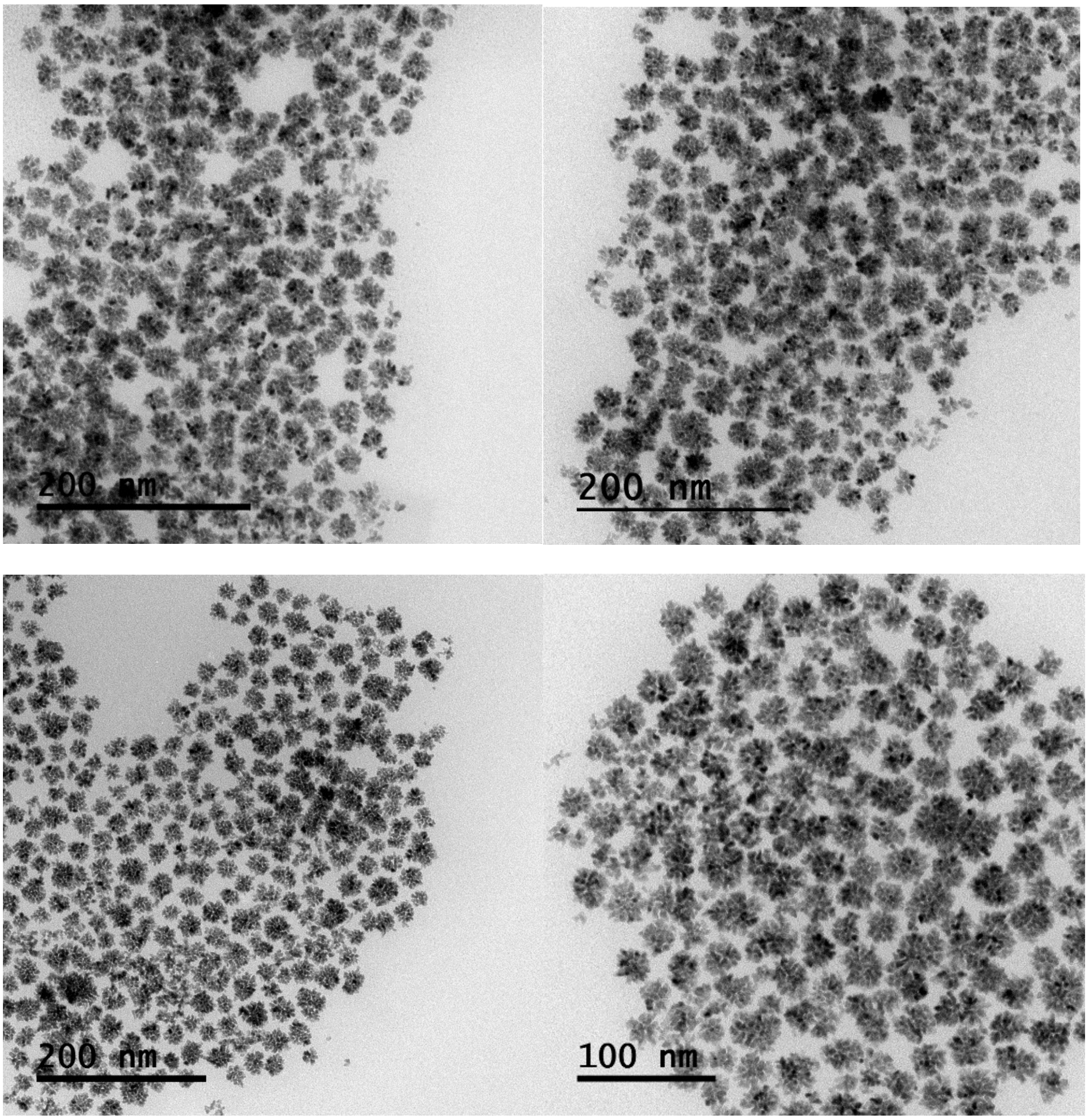
200 nm
200 nm
200 nm
100 nm

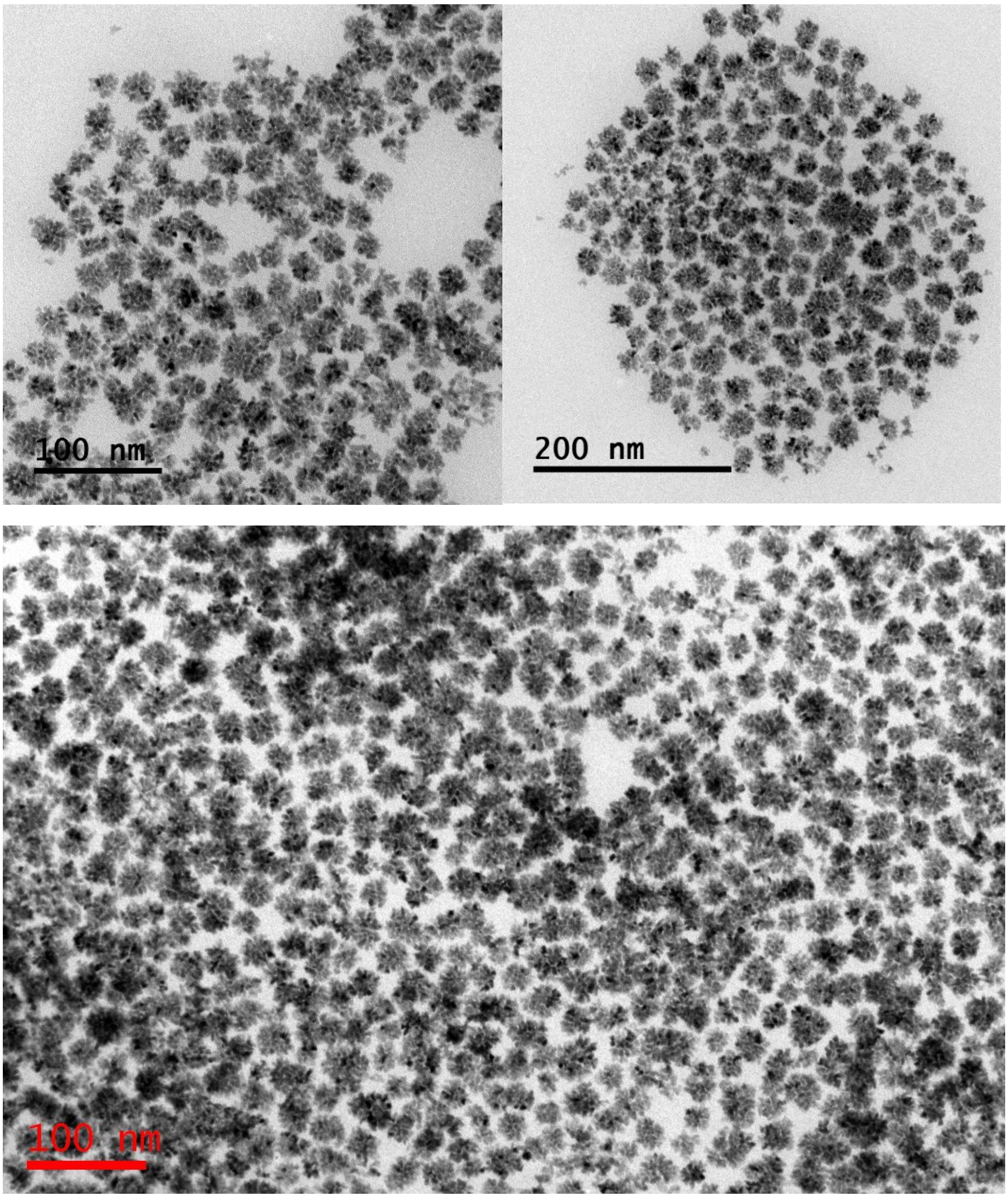

100 nm
200 nm
100 nm

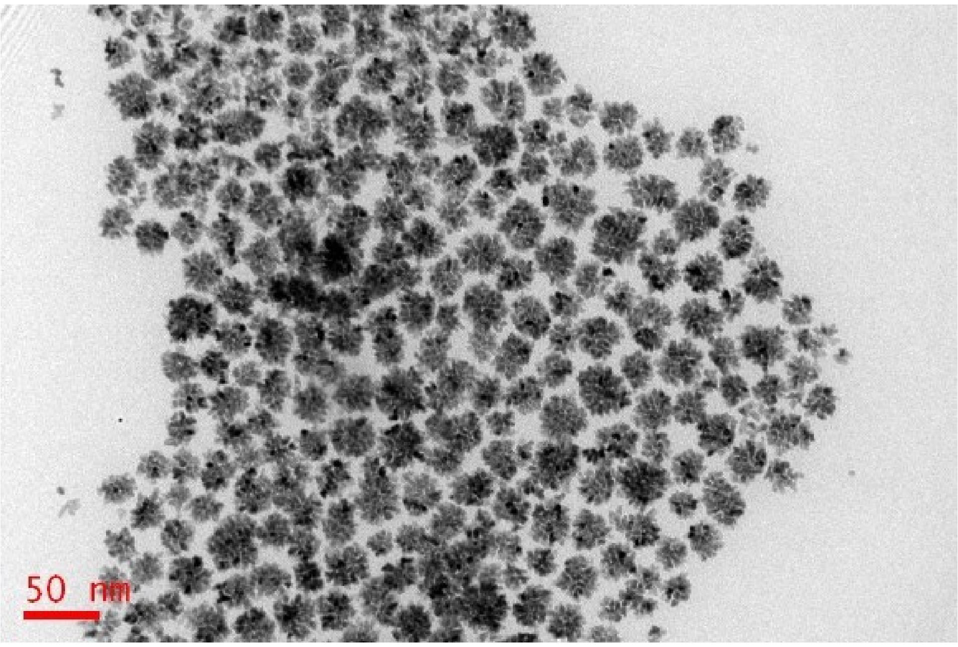
50 nm

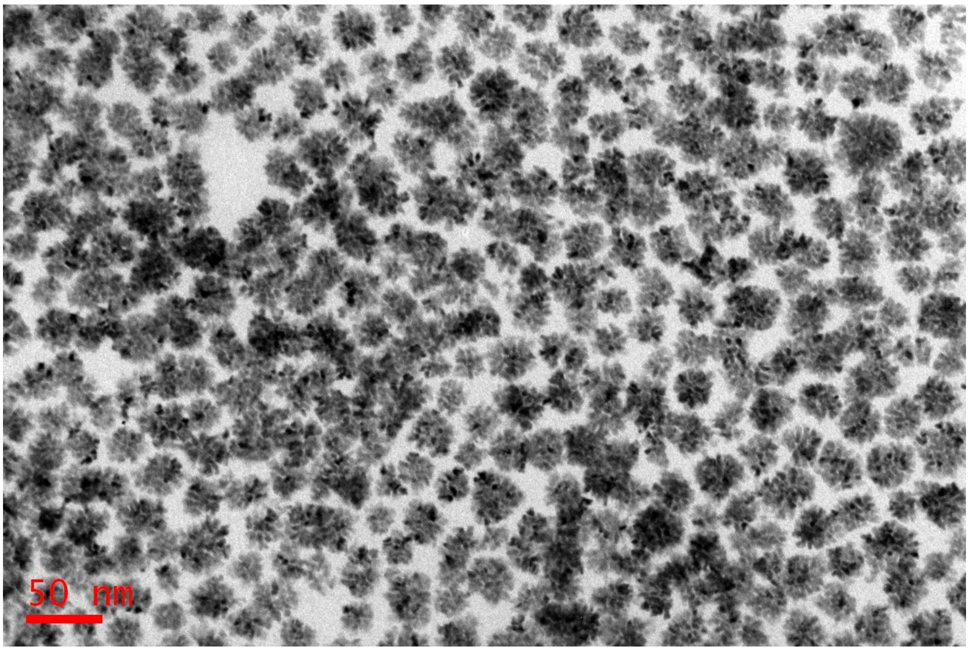
50 nm

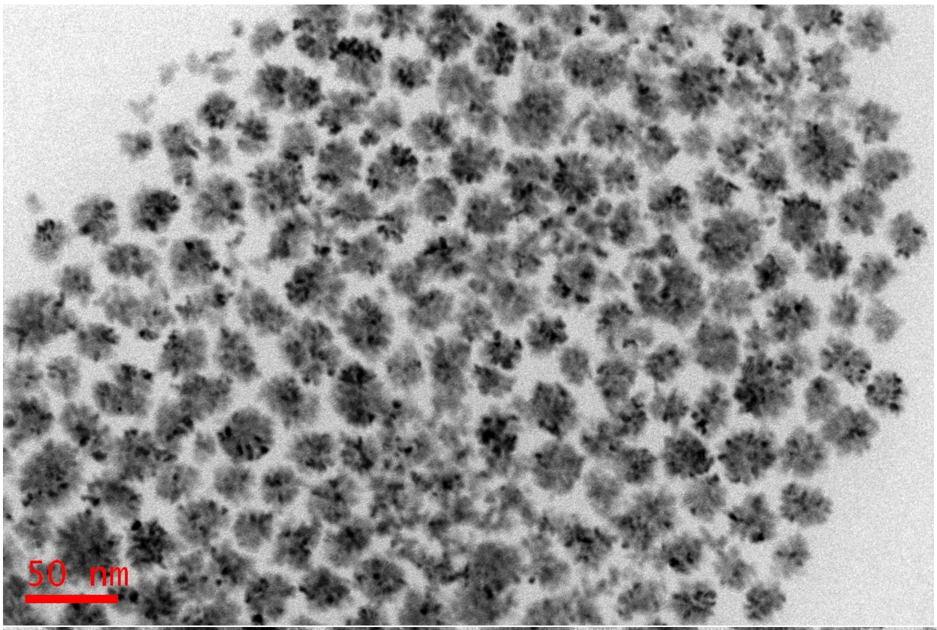
50 nm

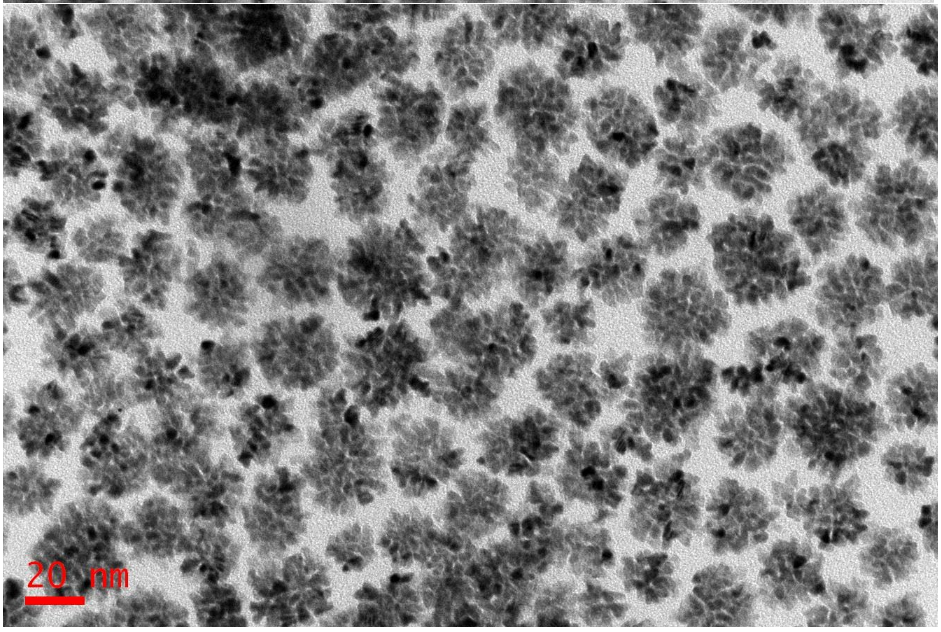
20 nm

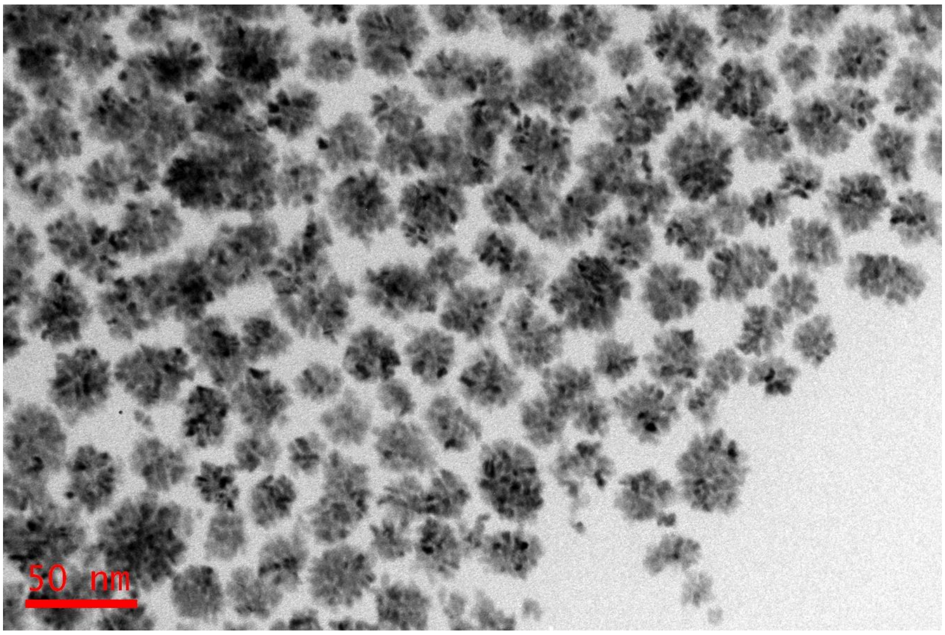

Fig. S1. TEM image of Cu-Pt nanodendrites measured at different positions and with different magnifications, demonstrating a highly branched structures of the synthesized samples.

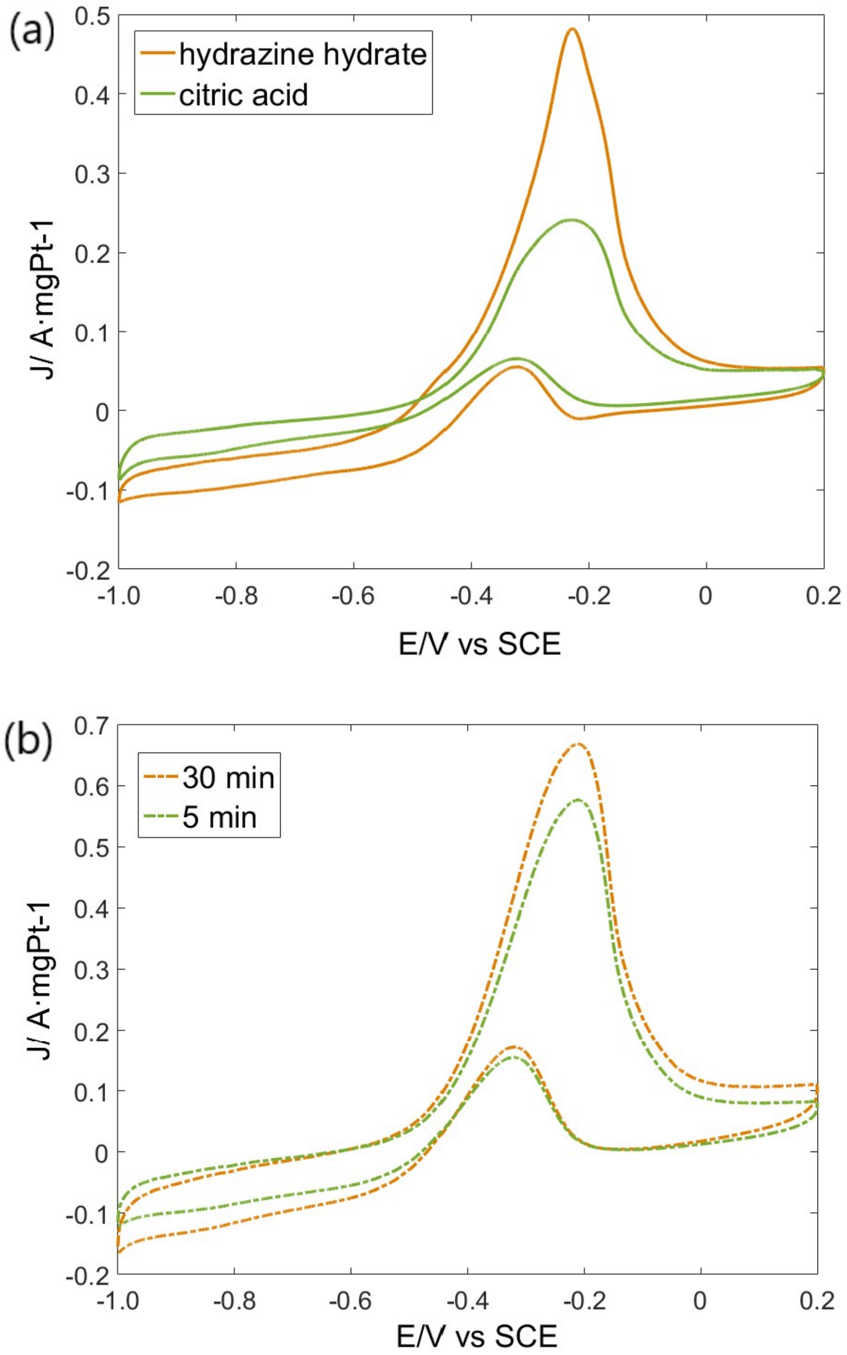

Fig. S2. Cyclic voltammetry (CV) curves of the products with different reducing agent (a), and with different reaction times (b) in 0.5 M NaOH and 0.5 M CH_3OH at a scan rate of 50 mV·s^{-1}.
